# Does Surgical Experience Affect the Outcomes During Percutaneous Release of the Trigger Finger?

**DOI:** 10.7759/cureus.46049

**Published:** 2023-09-27

**Authors:** Oğuzhan Çimen, Şahin Nami

**Affiliations:** 1 Department of Orthopaedics and Traumatology, Medistanbul Hospital, Istanbul, TUR; 2 Department of Orthopaedics and Traumatology, Avicenna Hospital, Istanbul, TUR

**Keywords:** steroid injection, diabetes, learning curve, percutaneous release, trigger fingers

## Abstract

Background

Trigger finger is a condition characterized by clicking or locking during finger movement, sometimes resulting in the freezing of a finger in flexion or extension. The aim of our retrospective study was to determine the effect of the surgeon’s learning curve on clinical outcomes in percutaneous release of the trigger finger. In addition, we evaluated the effects of diabetes and local steroid injections on clinical outcomes.

Methodology

A total of 954 trigger fingers in 678 patients were reviewed from 2012 to 2022. All percutaneous release procedures were performed by a single surgeon in our institute under local anesthesia. The main outcome measures were recurrence and patient satisfaction. In addition, all patients were evaluated in terms of re-operation and complications. The mean follow-up period was 54.87 months.

Results

There was complete relief of symptoms in 636 (93.81%) patients, and 22 (3.24%) patients had mild pain but were satisfied. We found that the success rate increased over time. The success rate was 91.4% in the first three years and increased to 98.25% in the next seven years (p = 0.001). There was no statistically significant difference between the diabetic and non-diabetic groups in terms of recurrence, satisfaction rate, and complications (p > 0.05). There was no statistically significant difference in terms of recurrence, satisfaction rate, and complications between the groups that received and did not receive steroid injections (p > 0.05).

Conclusions

Percutaneous release is a safe and reliable procedure in the treatment of trigger fingers, and the success rate increases as the experience increases. Moreover, diabetes mellitus and steroid injections did not affect the clinical results.

## Introduction

Trigger finger is a common condition that presents with strain, pain, and occasional locking during finger movements [1]. Surgical treatment can be considered when conservative treatments are unsuccessful. Trigger finger surgery can be performed either by traditional open surgery or by releasing the A1 pulley using a percutaneous technique [2]. The disadvantages of open release are the risk of infection, scarring, slower range of motion (ROM) recovery compared to the percutaneous technique, and higher cost [3-5], whereas the disadvantages of percutaneous release are iatrogenic digital nerve damage, tendon damage, incomplete release, and the possibility of conversion to open surgery [6-8]. However, anatomical studies specific to this procedure and increasing surgical experience have resolved these concerns over percutaneous release [9].

Percutaneous A1 pulley release has obvious advantages such as no open incisions, less scarring, less discomfort, shorter recovery, and lower cost [4,5]. Several studies have found comparable success and complication rates for open and percutaneous procedures, but shorter recovery rates consistent with the percutaneous technique [4,10,11].

Very few studies have evaluated the learning curve of the percutaneous technique. Previous studies have reported that the learning curve is short, surgeon success rates vary in individual years, and there is no clear trend in the learning curve [9,12].

In previous studies, diabetic and non-diabetic patients were compared in terms of recurrence and complication rates after percutaneous trigger finger release, and different results were reported [13,14]. The effect of local cortisone injection during percutaneous release is also unclear [15].

This large series study aimed to determine the effect of the surgeon’s learning curve on clinical outcomes in percutaneous release of the trigger finger. In addition, we evaluated the effects of diabetes and local steroid injections on clinical outcomes in patients who underwent percutaneous trigger finger release.

## Materials and methods

We performed a retrospective chart review of all outpatients based on percutaneous trigger finger release procedures performed between December 2012 and August 2022. A total of 773 patients underwent 1,084 trigger finger releases. The inclusion criteria were as follows: (1) being over 18 years of age, (2) failure of previous steroid injections into the flexor sheath, and (3) failure of conservative treatment for at least four months. We excluded patients who did not attend the final follow-up and had less than six months of follow-up, leaving 678 patients and 954 fingers for analysis.

Demographic information (age, gender, number of fingers operated), diabetes status, steroid injection status, and clinical findings were recorded by the orthopedic surgeon (SN).

Surgical techniques

All operations were done in an outpatient setting. All percutaneous release procedures were performed by a single surgeon (SN) at our institute under local anesthesia. The nodule was palpated while triggering, and with a marking pen, a 5-7 mm line was drawn. A1 pulley location may be different because of anatomical variation. Then, 1 ml of lidocaine hydrochloride (20 mg/mL) and epinephrine (0.0125 mg/mL) were injected subcutaneously beneath the drawn line. After several minutes, an 18-gauge hypodermic needle was inserted at a position with its sharp edge cuts, about 3-5 mm beneath the skin on the drawn line, and it was moved proximo-distally along the long axis of the tendon to cut the A1 pulley. Subsequently, the needle was inserted 3 mm away from the first portal, and the same movement was done. After withdrawing the needle, the patient was requested to flex and extend their digit, and triggering was checked. Triggering was resolved at this point for about 80% of patients. If there was any residual triggering, it was palpated again, mostly from the previous portals or rarely from a third portal. Again, triggering was checked, and if it was resolved, 0.25 mL betamethasone was injected into the wound. Steroid injection was not given to patients with severe hypertension and diabetes. To prevent infection and hematoma a bandage was wrapped until the next morning. Hand elevation was recommended for two days. No medication was prescribed. Patients could use their hands normally but were instructed to avoid manual work which applied pressure to the operated A1 pulley for three weeks.

Postoperatively, the patients were followed up in the outpatient clinic during the second week, third week, sixth month, and first year. At the last follow-up, patients were evaluated by phone and in the outpatient clinic. The main outcome measures were recurrence and patient satisfaction. Recurrence was defined as the persistence of the trigger. For patient satisfaction, the questionnaire applied in the study by Gilberts and Wereldsma was used [3]. The following questions were asked: Do you have triggering? Do you have pain? Do you have stiffness? Do you feel numbness? Do you have a scar? Are you dissatisfied, satisfied, or very satisfied with the treatment? In addition, all patients were evaluated for re-operation and all possible complications such as digital nerve injury, local tenderness, infection, and joint stiffness. If patients showed limited finger ROM, we performed additional finger rehabilitation. Our finger rehabilitation included passive hyperextension stretching of the metacarpophalangeal joint and proximal interphalangeal joint for 10 seconds at a time 30 times a course, and twice a day to restore full extension of the finger.

Statistical analysis

Continuous variables were expressed as means and standard deviation, and categorical variables were expressed as frequency and percentage. The chi-square test was used to analyze the categorical data distribution. Unpaired t-tests were used for comparing the means of independent groups. A p-value <0.05 was considered statistically significant.

## Results

A total of 678 patients with trigger fingers were included in the study. Overall, 427 were females and 251 were males. Of the 678 patients, 493 had triggering in a single finger, and 185 had triggering in more than one finger (two to seven fingers), for a total of 954 trigger fingers. The most frequently involved digit was the thumb (43.9%), followed by the ring, middle, little, and index fingers at 20.5%, 19.3%, 8.3%, and 8%, respectively. A total of 56 patients had diabetes mellitus (DM), 13 of whom had type 1 DM. During trigger finger percutaneous release, 522 patients received 0.25 mL of betamethasone (7 mg/mL) injection during the procedure (Table 1).

**Table 1:** Patient information.

Characteristic	Number (percentage)
Age (year)	52.8 ± 11.0
Gender	
Male	251 (37%)
Female	427 (63%)
Multiple trigger fingers	185
Affected digit	
Thumb	419 (43.9%)
Index finger	76 (8%)
Middle finger	184 (19.3%)
Ring finger	196 (20.5%)
Little finger	79 (8.3%)
Diabetes mellitus	56 (8.3%)
Release with steroid injection	522 (77%)

All patients were followed up at a mean of 54.87 months (range = 6-124 months). At the final clinical evaluation, 20 patients had a recurrence, whereby 15 patients underwent re-release. Thirteen of these re-releases were performed percutaneously and two were performed as open surgery. Five patients refused further re-releases. There was complete relief of symptoms (pain/triggering/locking) in 636 (93.81%) patients, and 22 (3.24%) patients had mild pain from time to time but were satisfied with the percutaneous release procedure (Table 2).

**Table 2:** Results after percutaneous release.

Characteristic	Patient, N	Fingers, N
Triggering	20 (3.0%)	22 (3.1%)
Pain	42 (6.2%)	47 (4.9%)
Stiffness	8 (1.2%)	9 (0.9%)
Digital nerve injury	0 (0%)	0 (0%)
Superficial wound infection	3 (0.4%)	3 (0.3%)
Scar	0 (0%)	0 (0%)
Satisfaction		
Dissatisfied	20 (3.0%)	
Satisfied	22 (3.2%)	
Very satisfied	636 (93.8%)	

**Figure 1:**
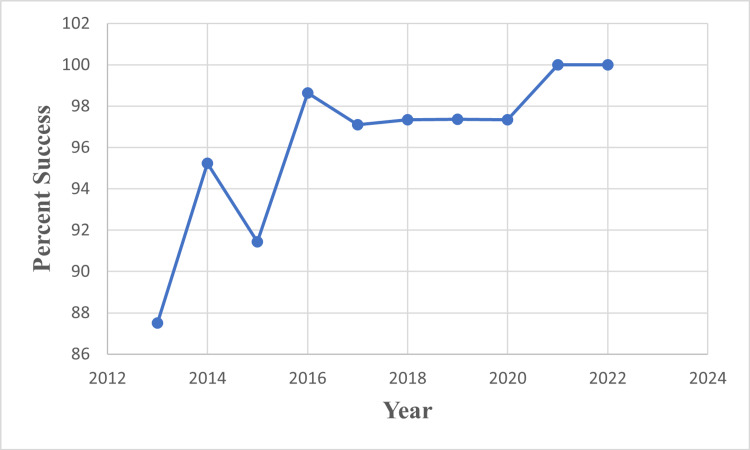
Percutaneous trigger finger release success rate. Very satisfied and satisfied patients evaluated it as a successful outcome.

We found that the surgeon’s success rate in individual years (very satisfied and satisfied patients) increased over time. While the average success rate was 91.4% in the first three years, this rate increased to 98.25% in the next seven years (p = 0.001) (Figure 1).

There was no statistically significant difference between the diabetic and non-diabetic groups in terms of recurrence, satisfaction rate, and complications (p > 0.05) (Table 3).

**Table 3:** Results of percutaneous release with or without DM. DM: diabetes mellitus; SD: standard deviation; NC: non-computable

Characteristic	DM (%)	Non-DM (%)	P-value
No.	56 (8.3%)	622 (91.7%)	
Mean age ± SD, yr	53.2 ± 11.7	52.7 ± 11.0	0.66
Female sex	34 (60.7%)	393 (63.2%)	0.71
Multiple trigger fingers	23 (41.1%)	162 (25.9%)	0.0009
Satisfaction			
Dissatisfied	1 (1.8%)	19 (3.1%)	0.68
Satisfied	2 (3.6%)	20 (3.2%)	
Very satisfied	53 (94.6%)	582 (93.5%)	
Complications			
Triggering	1 (1.8%)	19 (3.1%)	0.61
Pain	3 (5.4%)	39 (6.3%)	0.81
Stiffness	1 (1.8%)	7 (1.1%)	0.65
Digital nerve injury	0 (0%)	0 (0%)	NC
Wound infection	1 (1.8%)	2 (0.3%)	0.11
Scar	0 (0%)	0 (0%)	NC

There was no statistically significant difference in terms of recurrence, satisfaction rate, and complications between the groups that received and did not receive steroid injections during the percutaneous release procedure (p > 0.05) (Table 4).

**Table 4:** Results of percutaneous release with or without steroid injection. SD: standard deviation; NC: non-computable

Characteristic	Steroid group (%)	Non-steroid group (%)	P-value
No.	522 (77%)	156 (23%)	
Mean age ± SD, year	52 ± 11	55.3 ± 10.9	0.001
Female sex	323 (61.9%)	103 (66%)	0.35
Satisfaction			
Dissatisfied	16 (3.1%)	4 (2.6%)	0.95
Satisfied	16 (3.1%)	6 (3.8%)	
Very satisfied	490 (93.2%)	146 (93.6%)	
Complications			
Triggering	16 (3.1%)	4 (2.6%)	0.75
Pain	32 (6.1%)	10 (6.4%)	0.89
Stiffness	7 (1.3%)	1 (0.6%)	0.48
Digital nerve injury	0 (0%)	0 (0%)	NC
Wound infection	1 (0.2%)	2 (1.3%)	0.07
Scar	0 (0%)	0 (0%)	NC

The most common complications included pain (6.19%), triggering (2.95%), stiffness (1.18%), and superficial wound infection (0.44%).

## Discussion

Based on our retrospective study involving a large number of patients, percutaneous release is a safe and reliable procedure for the treatment of trigger finger, and the success rate increases as the surgeon’s experience increases. In addition, the presence of DM and whether steroid injection was applied during the percutaneous release procedure did not affect the clinical results. Many studies have reported similar results of open trigger finger surgery and percutaneous trigger finger release with successful outcome rates ranging from 90% to 100% [2,7,12,16-19]. Wang et al. [20] performed a meta-analysis using randomized trials and found no difference in failure rates or complication rates between open and percutaneous techniques.

Although Gil et al. [2] stated that the percutaneous technique requires a learning curve to reduce the risk of procedure-related complications, previous studies have reported that this technique is easy to learn and has a shorter learning curve [9,12]. Weiss et al. [9] performed percutaneous release on 596 trigger fingers (429 patients) over a 10-year period and reported no major complications with a success rate of 90.1%. They reported that the surgeon’s success rate in individual years varied and did not demonstrate a clear trend or learning curve for the procedure. In our study, we found that the success rate of the surgeon who performed percutaneous release was high even in the third year and increased in the following years.

Trigger finger is more common in diabetic patients compared to the general population. Their symptoms are more likely to be severe, affect multiple fingers, and have bilateral hand involvement [21]. The results of percutaneous trigger finger release are controversial in diabetic patients. Huang et al. [13] compared percutaneous A1 pulley release results between diabetic and non-diabetic patients and reported recurrent triggering in 15% of diabetic patients and only 5% of non-diabetic patients (p = 0.013). Although good results were obtained for both groups in the short term, diabetic patients reported worse long-term outcomes and worse satisfaction compared to non-diabetic patients. Abe [22] compared the results of percutaneous A1 pulley release and concomitant corticosteroid injection between diabetic and non-diabetic patients. At the final six-month follow-up, there was no difference in the percentage of satisfactory outcomes and clinical scores between non-diabetics and diabetics (96% vs. 92%). In our study, we found statistically significant multi-finger involvement in patients with diabetes. However, we observed that the satisfaction rate and complication rate of patients with diabetes and non-diabetic patients who underwent percutaneous trigger finger release were similar.

The addition of local steroid injections during percutaneous trigger finger release is controversial. Patel et al. [7] in a prospective cohort study compared percutaneous release outcomes when used alone or in combination with steroid injections and found a success rate of 89% (92/105) in the non-steroid group and 96% (115/120) in the steroid group (p = 0.040). However, a meta-analysis of 34 studies which included 2,114 trigger fingers reported that the pooled success rates were 94.3% (454/481) in the steroid group and 94.9% (1,550/1,633) in the nonsteroid group (p = 0.650) [23]. Jegal et al. [24] performed a randomized trial to determine whether adding a steroid injection after percutaneous trigger finger release would improve outcomes. In this study, which included 91 patients, they found that steroid injection during percutaneous release reduced pain and improved subjective outcomes in the early postoperative period (third week); however, these effects did not persist at three months. Liu et al. [15] performed a study to determine whether adding a steroid injection after percutaneous trigger finger release would improve outcomes. In this study, which included 432 trigger fingers, they reported that the success rate at three months was 98.4% and there was no difference between the groups that received and did not receive steroids. However, extensor lag was statistically significantly lower in those who received steroids compared to those who did not (5.4% vs. 12.7%) during the first week. These studies show us that the addition of cortisone injection during percutaneous relaxation is beneficial in the early term, but not in the long term. In our study, we observed that it had no effect on the long-term results.

There are some limitations of this study. It is a retrospective study, all surgical procedures were performed by a single surgeon, and the final follow-up of some patients was done only by telephone.

## Conclusions

Percutaneous release is a safe and reliable procedure in the treatment of trigger finger, and the success rate increases as the surgeon’s experience increases. In addition, the presence of DM and steroid injections do not affect the clinical results.
